# Detection of oriented fractal scaling components in anisotropic two-dimensional trajectories

**DOI:** 10.1038/s41598-020-78807-z

**Published:** 2020-12-14

**Authors:** Ivan Seleznov, Anton Popov, Kazuhei Kikuchi, Elena Kolosova, Bohdan Kolomiiets, Akio Nakata, Miki Kaneko, Ken Kiyono

**Affiliations:** 1 Graduate School of Engineering Science, Osaka University, Toyonaka, 560-8531 Japan; 2 Department of Electronic Engineering, Igor Sikorsky Kyiv Polytechnic Institute, Kyiv, 03056 Ukraine; 3 Ciklum Data & Analytics, Kyiv, Ukraine; 4 School of Statistical Thinking, Institute of Statistical Mathematics, Tokyo, 190-8562 Japan; 5 Scientific Research Institute, National University of Physical Education and Sport of Ukraine, Kyiv, 03150 Ukraine; 6 Development Department, Union Tool Co., Tokyo, 140-0013 Japan

**Keywords:** Geophysics, Statistical physics, thermodynamics and nonlinear dynamics, Nonlinear phenomena, Bone quality and biomechanics

## Abstract

We propose a novel class of mixed fluctuations with different orientations and fractal scaling features as a model for anisotropic two-dimensional (2D) trajectories hypothesized to appear in complex systems. Furthermore, we develop the oriented fractal scaling component analysis (OFSCA) to decompose such mixed fluctuations into the original orientation components. In the OFSCA, the original orientations are detected based on the principle that the original angles are orthogonal to the angles with the minimum and maximum scaling exponents of the mixed fluctuations. In our approach, the angle-dependent scaling properties are estimated using the Savitzky–Golay-filter-based detrended moving-average analysis (DMA), which has a higher detrending order than the conventional moving-average-filter-based DMA. To illustrate the OFSCA, we demonstrate that the numerically generated time-series of mixed fractional Gaussian noise (fGn) processes with non-orthogonal orientations and different scaling exponents is successfully decomposed into the original fGn components. We demonstrate the existence of oriented components in the 2D trajectories by applying OFSCA to real-world time-series, such as human postural fluctuations during standing and seismic ground acceleration during the great 2011 Tohoku-oki earthquake.

## Introduction

Noise and fluctuations frequently display fractal-like scaling properties associated with long-range correlations in real-world complex systems, such as biological, geophysical, and economical systems^1,2^. The appearance of such fluctuations has been considered as a key marker associated with a universal principle hidden in the complex system dynamics^3^. Hence, various analysis methodologies have been developed to provide a detailed characterisation of such complex fluctuations. These methods have demonstrated that the scaling properties emerge not only in an auto-correlation of the time series^4–6^, but also in higher moment correlation (e.g. multi-fractality)^7–9^ and in a cross-correlation between multivariate time series^10,11^.

**Figure 1:**
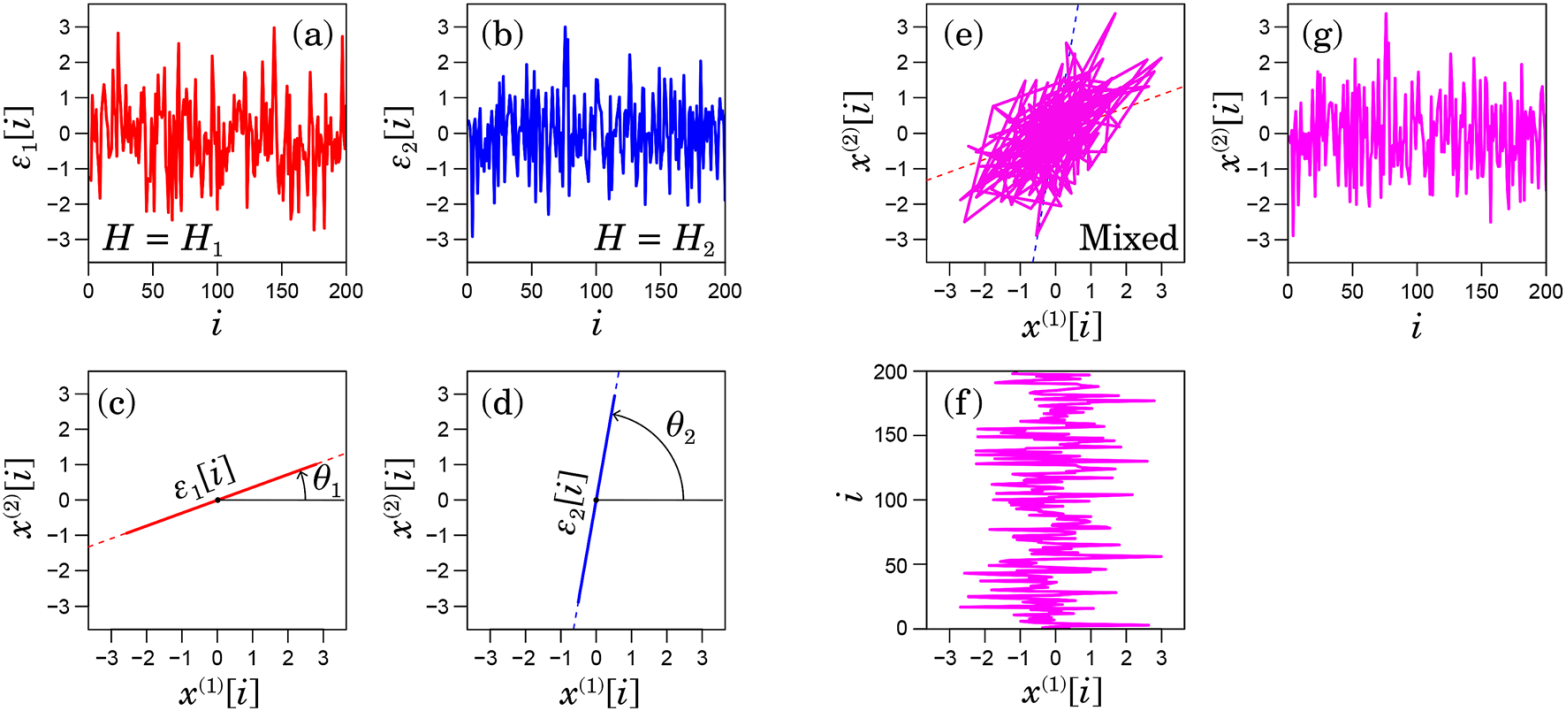
Illustration of a mixed fGn model with $H_1 = 0.8$, $\theta _1 = \pi /6$, $H_2 = 0.6$, and $\theta _1 = 4\pi /9$. (**a**,**b**) Original components $\{\epsilon _{1}[i]\}$ and $\{\epsilon _{2}[i]\}$. (**c**,**d**) The orientations of $\{\epsilon _{1}[i]\}$ and $\{\epsilon _{2}[i]\}$ in $(x^{(1)}, x^{(2)})$ plane. (**e**–**g**) Mixed fluctuations of $\{\epsilon _{1}[i]\}$ and $\{\epsilon _{2}[i]\}$ using Eq. (6) in the $(x^{(1)}, x^{(2)})$ plane.

The fractal scaling behaviour in two-dimensional (2D) trajectories is also of interest in real-world applications. For instance, human postural fluctuations during quiet standing^12^ and animal movement patterns^13^ have been modelled using 2D fractional Brownian motion (fBm) and fractional Gaussian noise (fGn). Most of the previous studies have characterised the 2D trajectories using the projections onto the orthogonal directions and otherwise under the assumption of 2D isotropic properties^12,13^. Such approaches cannot provide any information on geometrically anisotropic structure of the system. In this study, we hypothesise that real-world 2D trajectories display anisotropy that is different from conventional, isotropic 2D fBm and fGn^14^. Moreover, we propose a novel class of mixed fluctuations with different orientations and scaling features as a model for anisotropic 2D trajectories. In our model, the mixed fluctuations are assumed to consist of two independent components with non-orthogonal orientations as shown in Fig. 1. Such anisotropy has not yet been systematically investigated. The open problem here is to detect the hidden orientation components in the observed trajectories. To solve this problem, we develop the oriented fractal scaling component analysis (OFSCA) to characterise the directional properties of such trajectories and decompose the mixed fluctuations into the original components. In the OFSCA, the original orientations are detected based on the principle that the original angles are orthogonal to the angles with the minimum and maximum scaling exponents of the mixed fluctuations.

In our approach, we introduce an extension of the detrended moving-average analysis (DMA)^15^: the directional DMA (DDMA). The purpose of DDMA is to estimate the Hurst scaling exponent associated with the fBm- and fGn-like property in the projection of the observed time-series onto an axis forming angle $\theta$ with the abscissa axis. In real-world time-series analysis, it has been shown that non-stationary trends embedded in the time series harmfully affect the scaling exponent estimation and induce a misinterpretation of the correlation properties^16^. Therefore, scaling analysis methods such as detrending procedures have been widely used instead of conventional Fourier power spectral, rescaled range^4^, and structure-function^17^ analyses. For instance, the practical options are the wavelet-decomposition-based method with a wavelet having vanishing moments^5^ and detrending-operation-based scaling analysis methods, such as detrended fluctuation analysis (DFA)^16,18^ and DMA^19^. Recently, we have established the theoretical foundation for DMA including higher-order DMA^6,15,20^ and developed a fast implementation algorithm for DMA^21^ to increase the reliability and applicability of DMA. In this study, we further extend the application of DMA to 2D trajectory analysis.

The remainder of this paper is organised as follows. First, we introduce the analysis procedure of anisotropic 2D trajectories. We illustrate our approach using a numerical sample, assuming a mixed fGn model displaying anisotropic long-range correlations. We then analyse real-world time-series, such as human postural fluctuations during standing and seismic ground acceleration during the great 2011 Tohoku-oki earthquake and discuss the interpretation of the oriented scaling components in the 2D trajectories. Finally, we provide a summary and some perspectives on future research.

**Figure 2:**
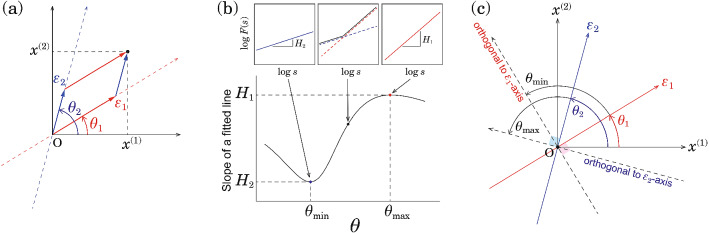
Principle illustration of the orientation detection of angle-dependent long-range correlated components $\{\epsilon _{1}[i]\}$ and $\{\epsilon _{2}[i]\}$ in the $(x^{(1)}, x^{(2)})$ plane. (**a**) Relation between $(x_{1}, x_{2})$ and $(\epsilon _{1}, \epsilon _{2})$. (**b**) $\theta$-dependent slope estimation of $\log _{10} F^{(\theta )} (s)$ vs. $\log _{10} s$. (**c**) Relation between minimum and maximum slope angles $(\theta _{\mathrm{min}}, \theta_{\mathrm{max}})$ and original component angles $(\theta _{1}, \theta _{2})$.

## Analysis method for anisotropic 2D trajectories

Let us consider a 2D time-series (trajectory) represented as the ordered series of points in the Cartesian coordinate plane, $\left\{ (x^{(1)}[i], x^{(2)}[i])\right\}$ ($i = 1, 2, \ldots N$, where *N* is the length of the time series). If the time series is a sample path of 2D fGn given by two independent fGn sample paths, $\left\{ x^{(1)}[i]\right\}$ and $\left\{ x^{(2)}[i]\right\}$, which are two series of fBm increments with the same Hurst exponent *H*, the auto-correlation properties are isotropic, independent of the orientation. Thus, the scaling property of each angular component (projection onto a rotated axis) is identical and not affected by any rotational transform. We introduce methods for detailed anisotropic characterisation assuming an anisotropic 2D time-series different from such 2D fGn. In our approach, we first evaluate the angle-dependent scaling properties using higher-order DMA^21^ (henceforth, called DDMA) and then decompose the observed 2D time series into two components with different orientations and scaling properties.

### Angle-dependent scaling analysis

The observed time series is projected onto an axis forming angle $\theta$ with the positive direction of the abscissa axis to detect the anisotropic scaling behaviour. Projected time series $\left\{ x^{(\theta )} [i]\right\}$ is given as

1 $$\begin{aligned} x^{(\theta )} [i] = x^{(1)}[i] \, \cos \theta + x^{(2)}[i] \, \sin \theta , \end{aligned}$$

where $\theta$ is varied across the range of $0 \le \theta < \pi$.

Time series $\left\{ x^{(\theta )} [i]\right\}$ for each $\theta$ is analysed using the Savitzky–Golay-filter-based DMA^6,22^. In the DDMA, we first calculate the integrated series of $\left\{ x^{(\theta )} [i]\right\}$.

2 $$\begin{aligned} y^{(\theta )} [i] = \sum _{j=1}^{i} x^{(\theta )} [j] . \end{aligned}$$

Note that, if $\left\{ x^{(\theta )} [i]\right\}$ is an fBm trajectory, we can skip this integration procedure and analyse $\left\{ x^{(\theta )} [i]\right\}$ instead of $\left\{ y^{(\theta )} [i]\right\}$ in the following calculation.

We then calculate the directional fluctuation function, $F^{(\theta )}(s)$, defined as

3 $$\begin{aligned} F^{(\theta )} (s) = \sqrt{ \frac{1}{N - s + 1} \sum _{i= (s+1)/2}^{ N - (s-1)/2} \left( y^{(\theta )} [i] - \widetilde{y}_{\mathrm{SG}}^{(m,s)}[i] \right) ^2 }, \end{aligned}$$

where $\widetilde{y}_{\mathrm{SG}}^{(m,s)}$ represents the smoothed version of $y^{(\theta )} [i]$ obtained after applying the Savitzky–Golay filter with a polynomial of degree *m* and window length *s*. In Eq. (3), the signals $\left\{ \widetilde{y}_{\mathrm{SG}}^{(m,s)} [i]\right\}$ locally smoothed using the Savitzky–Golay filter are removed from the time series^22^ to attenuate the baseline non-stationarity embedded in the observed time series $\left\{ y^{(\theta )} [i]\right\}$. The Savitzky–Golay filter smoothes a noisy signal by adjusting a piecewise polynomial function with degree *m* to the signal and can remove the polynomial trends up to the order $(m + 1)$.

Anisotropic properties can be evaluated using the $\theta$-dependent heterogeneity in $F^{(\theta )} (s)$. In addition, the long-range correlation observed in each orientation, $\theta$, is evaluated using the power-law increase of $F^{(\theta )} (s)$ as

4 $$\begin{aligned} F^{(\theta )} (s) \sim s^{\alpha (\theta )}, \end{aligned}$$

and quantified using scaling exponent $\alpha (\theta )$ estimated as the slope of the double-logarithmic plot of $F^{(\theta )} (s)$ against *s*.

Note that $F^{(\theta )}(s)$ can be directly linked with auto-correlation function $C^{(\theta )} (k)$ and power spectrum $S^{(\theta )}(f)$ of $\left\{ x^{(\theta )} [i] \right\}$^11,23^. That is, we have

5 $$\begin{aligned} F^{(\theta )} (s) = \sqrt{\sum _{k=-s}^{s} C^{(\theta )} (k)\, L(k, s)} = \sqrt{\int _{-1/2}^{1/2} \left| S^{(\theta )}(f)\right| \left| G_s(f) \right| ^2 df}. \end{aligned}$$

The analytical forms of kernels *L*(*k*, *s*) and $\left| G_s(f) \right| ^2$ were demonstrated in^11,23^. Using these relations and assuming Eq. (4), we can show the scaling relations, $\alpha = 1-\gamma /2$ when $C^{(\theta )} (k) \sim k^{-\gamma }$ ($0< \gamma < 1$) and $\alpha = (\beta +1)/2$ when $S^{(\theta )} (f) \sim f^{-\beta }$ ($-1< \beta < 2 m + 3$). In particular, when $C^{(\theta )}(k)$ decays exponentially to zero and $S^{(\theta )}(f)$ shows the low-frequency plateau indicating short-term correlation, the scaling exponent results asymptotically in $\alpha = 0.5$.

In a previous study^11^, it was analytically shown that time-scale distortion between the scale in the time domain of DMA and the frequency in the Fourier spectral domain is induced by the higher-order DMA. Thus, we use the corrected time scale, $\tilde{s}$, instead of *s*. Although scale *s* in the zeroth-order DMA corresponds well to frequency *f* in the Fourier spectral domain, i.e., $\tilde{s}=s/1.00$, $\tilde{s}$ are given by $\tilde{s}=s/1.93$ and $\tilde{s}=s/2.74$ in the second- and fourth-order DMAs, respectively. Note that a straightforward implementation of the aforementioned procedure has a high computational complexity. Thus, a fast algorithm of DMA should be employed in the practical analysis^21^.

**Figure 3:**
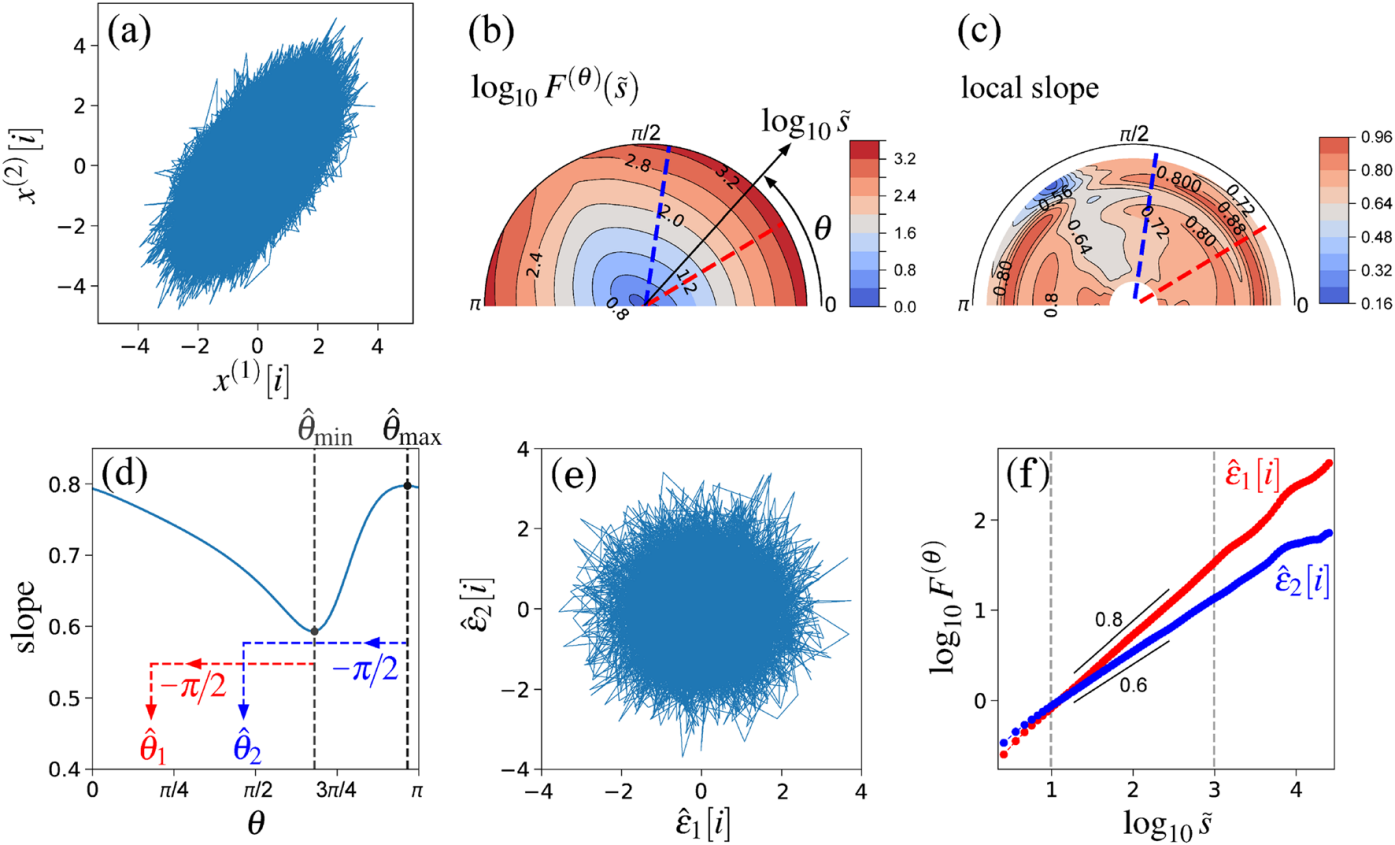
Orientation decomposition of the mixed fGn model (Eq. (6)) with $H_1=0.8$, $\theta _1=\pi /6=30^{\circ }$, $H_2=0.6$, and $\theta _2=4\pi /9=80^{\circ }$. (**a**) Sample path of $(x_{1}, x_{2})$. (**b**) Angle dependence of $\log _{10} F^{(\theta )} (\tilde{s})$ vs. $\log _{10} \tilde{s}$, where $\tilde{s}=s/1.93$ in the second-order DDMA. (**c**) Angle dependence of the local slopes of $\log _{10} F^{(\theta )} (\tilde{s})$ vs. $\log _{10} \tilde{s}$. (**d**) Angle dependence of the slope in the range of $1< \log _{10} \tilde{s} < 3$. (**e**) Reconstructed components $(\hat{\epsilon }_{1}, \hat{\epsilon }_{2})$. (**f**) Fluctuation functions of the reconstructed components of $\hat{\epsilon }_{1}$ with $\theta _{1} = 31^{\circ }$ and $\hat{\epsilon }_{2}$ with $\theta _{2} = 82^{\circ }$; the slopes in the range of $1< \log _{10} \tilde{s} < 3$ were 0.8 and 0.6, respectively, in the second-order DDMA.

### Decomposition of mixed long-range correlated fluctuations

We introduce a new class of 2D long-range correlated processes displaying special orientations and an orientation decomposition method for such processes. We illustrate our approach by assuming a mixed fGn model displaying anisotropic long-range correlations (Fig. 1). Our model consists of two independent fGn processes $\{\epsilon _{1}[i]\}$ and $\{\epsilon _{2}[i]\}$ with two different Hurst exponents $H_1$ and $H_2$ ($H_{1} > H_{2}$), respectively (Fig. 1a,b). It mixes these processes oriented at angles $\theta _1$ and $\theta _2$ to the *x*-axis (Fig. 1c,d) as

6 $$\begin{aligned} \begin{bmatrix} x^{(1)}[i]\\ x^{(2)}[i] \end{bmatrix} = \begin{bmatrix} \cos \theta _1 &{} \cos \theta _2\\ \sin \theta _1 &{} \sin \theta _2 \end{bmatrix} \begin{bmatrix} \epsilon _{1}[i]\\ \epsilon _{2}[i] \end{bmatrix}. \end{aligned}$$

Now, we consider the problem of decomposing the observation of $\left\{ \left( x^{(1)}[i], x^{(2)}[i]\right) \right\}$ (Fig. 1e,g,f) into the original fGn time series, $\{\epsilon _{1}[i]\}$ and $\{\epsilon _{2}[i]\}$. Notably, the independent component analysis (ICA) is not properly applicable to our model, because both $x^{(1)}[i]$ and $x^{(2)}[i]$ in our model follow Gaussian distributions. Two linearly mixed independent Gaussian processes cannot be separated uniquely into two independent components (ICA for Gaussian processes is equivalent to principal component analysis).

Our approach is described as follows (Fig. 2). In this model [Eq. (6)], the projected time series is given by

7 $$\begin{aligned} x^{(\theta )}[i] = \epsilon _{1}[i] \cos \left( \theta - \theta _1 \right) + \epsilon _{2}[i] \cos \left( \theta - \theta _2 \right) . \end{aligned}$$

This equation indicates that, when $\theta = \theta _1 \pm \pi /2$, $x^{(\theta )} [i]$ is orthogonal and independent of $\epsilon _{1}[i]$ (see Fig. 2c). That is, we obtain

8 $$\begin{aligned} x^{(\theta _1 + \pi /2)}[i]= \epsilon _{2}[i] \cos \left( \theta _1 - \theta _2 + \pi /2 \right) , \end{aligned}$$

which is proportional to the original $\epsilon _{2}[i]$ with $H_2$. For the same reason, $x^{(\theta _2 \pm \pi /2)} [i]$ is orthogonal to $\epsilon _{2}[i]$ and proportional to $\epsilon _{1}[i]$ with $H_1$. In contrast, when $\theta \ne \theta _1 \pm \pi /2$ and $\theta \ne \theta _2 \pm \pi /2$, $F^{(\theta )}(s)$ shows a crossover of scaling exponents (see Fig. 2b). The forced linear fit to the broken lines in the log–log plot of $F^{(\theta )} (s)$ vs. *s* yields a slope in the range $(H_1, H_2)$. Therefore, seeking two main orientations, $\hat{\theta }_{\mathrm{min}}$ and $\hat{\theta }_{\mathrm{max}}$, respectively, with the minimum and maximum values of $\alpha (\theta )$, we can estimate the original orientations of $\epsilon _{1}[i]$ and $\epsilon _{2}[i]$ as $\hat{\theta }_1 = \hat{\theta }_{\mathrm{min}} \pm \pi /2$ and $\hat{\theta }_2 = \hat{\theta }_{\mathrm{max}} \pm \pi /2$, respectively. In this paper, the hat denotes an estimate of the corresponding quantity. Using the angles $\hat{\theta }_1$ and $\hat{\theta }_2$, we can estimate the original signals as

9 $$\begin{aligned} \begin{bmatrix} \hat{\epsilon }_{1}[i]\\ \hat{\epsilon }_{2}[i] \end{bmatrix} = \begin{bmatrix} {\sin \hat{\theta }_2}/{\sin \left( \hat{\theta }_2 - \hat{\theta }_1\right) } &{} {\cos \hat{\theta }_2}/{\sin \left( \hat{\theta }_1 - \hat{\theta }_2\right) }\\ {\sin \hat{\theta }_1}/{\sin \left( \hat{\theta }_1 - \hat{\theta }_2\right) } &{} {\cos \hat{\theta }_1}/{\sin \left( \hat{\theta }_2 - \hat{\theta }_1\right) } \end{bmatrix} \begin{bmatrix} x^{(1)}[i]\\ x^{(2)}[i] \end{bmatrix} . \end{aligned}$$

We refer to this approach as the OFSCA.

### Numerical test

We analyse the sample time series (Fig. 3a) of the mixed fGn model with $H_1 = 0.8$, $\theta _1=\pi /6 = 30^{\circ }$, $H_2 = 0.6$, and $\theta _1=4 \pi /9 = 80^{\circ }$ as a numerical demonstration of the OFSCA. The angle dependence of $F^{(\theta )} (\tilde{s})$ is evaluated over the range of $0 \le \theta < \pi$ in increments of $\pi /64$ rad. The estimated $F^{(\theta )}(\tilde{s})$ are plotted in a circular cylindrical coordinate $(\rho , \phi , z)=(\log _{10} \tilde{s}, \theta , \log _{10} F^{(\theta )}(\tilde{s}))$, where $\rho$ is the distance of a coordinate point from the Cartesian *z*-axis, and $\phi$ is its azimuthal angle. In Fig. 3b, $\log _{10} F^{(\theta )}(\tilde{s})$ are plotted using the colour scale. In addition, the local slopes of $\log _{10} F^{(\theta )}(\tilde{s})$ vs. $\log _{10} \tilde{s}$ at each $\theta$ are plotted in Fig. 3c.

Although Fig. 3b,c show anisotropic scaling properties, the apparent orientations are observed in the major and minor axes of the ellipse-shaped fluctuations in Fig. 3a. Therefore, the conventional principal component analysis cannot decompose the observed time-series into the original orientations. In contrast, our approach makes it possible to estimate the original orientations of the two component time-series and to reconstruct the original time-series. In our approach, we first estimate $\hat{\theta }_{\mathrm{min}} = 121^{\circ }$ and $\hat{\theta }_{\mathrm{max}} = 172^{\circ }$ based on the angle dependence of the least-squares-fit slope as shown in Fig. 3d. Using Eq. (9) with $\hat{\theta }_1=\hat{\theta }_{\mathrm{min}} - 90^{\circ } = 31^{\circ }$ and $\hat{\theta }_2=\hat{\theta }_{\mathrm{max}} - 90^{\circ } = 82^{\circ }$, we decompose $\left\{ \left( x^{(1)}[i], x^{(2)}[i]\right) \right\}$ into $\{\hat{\epsilon }_{1}[i]\}$ and $\{\hat{\epsilon }_{2}[i]\}$ (Fig. 3e). The isotropic scattering behaviour in Fig. 3f indicates that the decomposed $\{\hat{\epsilon }_{1}[i]\}$ and $\{\hat{\epsilon }_{2}[i]\}$ are well uncorrelated with each other. The estimated scaling exponents of $\{\hat{\epsilon }_{1}[i]\}$ and $\{\hat{\epsilon }_{2}[i]\}$ reproduced the theoretical values (Fig. 3f).

## Real-world anisotropic scaling

**Figure 4:**
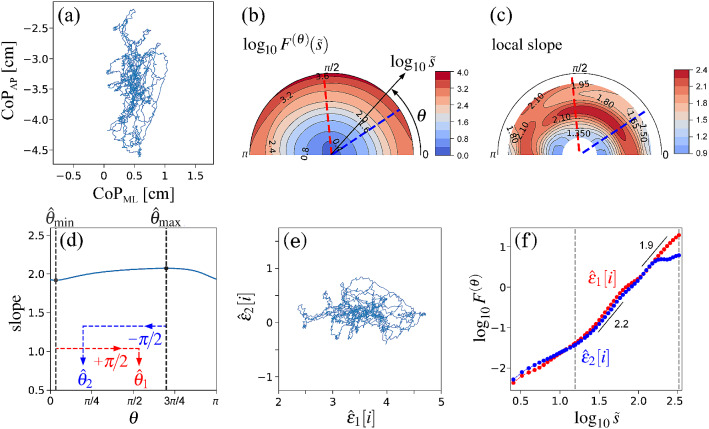
Orientation decomposition of centre-of-pressure (CoP) trajectory of a subject performing quiet standing with eyes-open condition. (**a**) Anteroposterior (AP) CoP vs. Mediolateral (ML) CoP. (**b**) Angle dependence of $\log _{10} F^{(\theta )} (\tilde{s})$ vs. $\log _{10} \tilde{s}$, where $\tilde{s}=s/1.93$ in the second-order DDMA. (**c**) Angle dependence of the local slopes of $\log _{10} F^{(\theta )} (\tilde{s})$ vs. $\log _{10} \tilde{s}$. (**d**) Angle dependence of the slope in the range of $1.2< \log _{10} \tilde{s} < 2.5$. (**e**) Reconstructed components $(\hat{\epsilon }_{1}, \hat{\epsilon }_{2})$. (**f**) Fluctuation functions of the reconstructed components of $\hat{\epsilon }_{1}$ with $\theta _{1} = 96^{\circ }$ and $\hat{\epsilon }_{2}$ with $\theta _{2} = 34^{\circ }$ in second-order DDMA.

**Figure 5:**
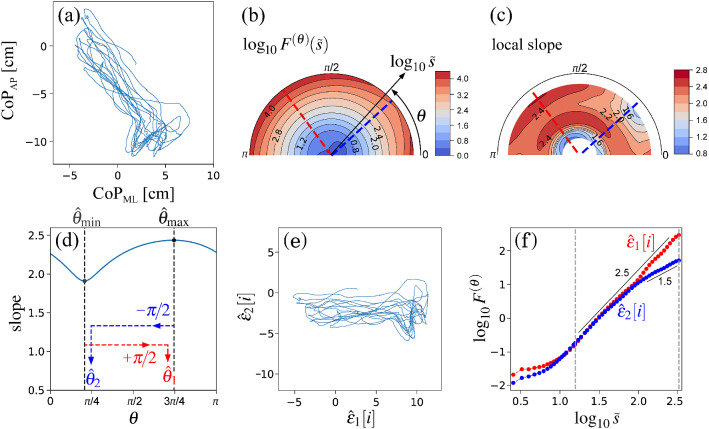
Orientation decomposition of the CoP trajectory of a subject performing a voluntary sway movement from the steady position to right-backward position with eyes open. (**a**) AP CoP vs. ML CoP. (**b**) Angle dependence of $\log _{10} F^{(\theta )} (\tilde{s})$ vs. $\log _{10} \tilde{s}$, where $\tilde{s}=s/1.93$ in the second-order DDMA. (**c**) Angle dependence of the local slopes of $\log _{10} F^{(\theta )} (\tilde{s})$ vs. $\log _{10} \tilde{s}$. (**d**) Angle dependence of the slope in the range of $1.2< \log _{10} \tilde{s} < 2.5$. (**e**) Reconstructed components $(\hat{\epsilon }_{1}, \hat{\epsilon }_{2})$. (**f**) Fluctuation functions of the reconstructed components of $\hat{\epsilon }_{1}$ with $\theta _{1} = 127^{\circ }$ and $\hat{\epsilon }_{2}$ with $\theta _{2} = 42^{\circ }$ in second-order DDMA.

**Figure 6:**
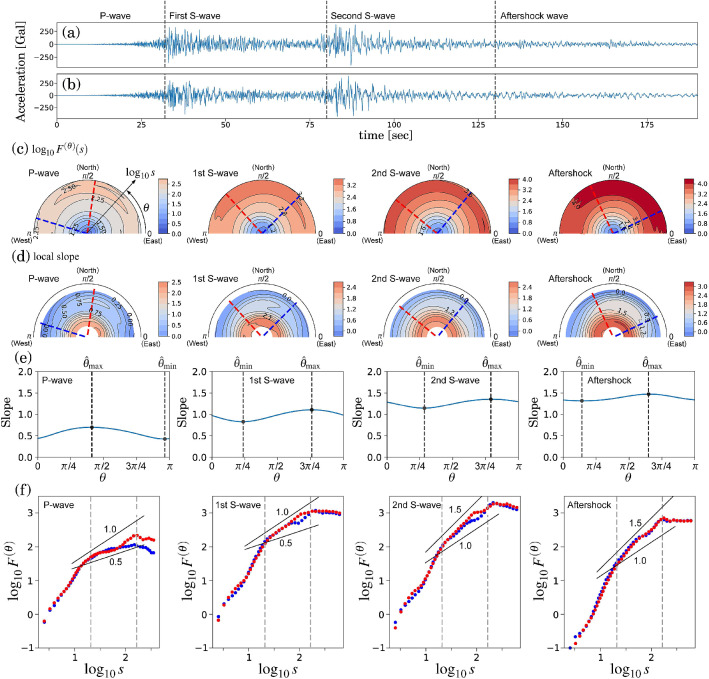
Orientation decomposition of the earthquake ground acceleration. Details were described in the text. (**a**,**b**) Ground acceleration with the north–south (**a**) and east–west (**b**) axes. (**c**) Angle dependence of $\log _{10} F^{(\theta )} (\tilde{s})$ vs. $\log _{10} \tilde{s}$, where $\tilde{s}=s/2.74$ in fourth-order DDMA. (**d**) Angle dependence of the local slopes of $\log _{10} F^{(\theta )} (\tilde{s})$ vs. $\log _{10} \tilde{s}$. (**e**) Angle dependence of the slope in the range of $1.3< \log _{10} \tilde{s} < 2.2$. (**f**) Fluctuation functions of the reconstructed components of $\hat{\epsilon }_{1}$ and $\hat{\epsilon }_{2}$ in fourth-order DDMA.

We demonstrate the existence of anisotropic scaling by analysing real-world time-series, such as human postural fluctuations during standing (Figs. 4 and 5) and seismic ground acceleration during the great 2011 Tohoku-oki earthquake (Fig. 6).

Python source code for computing OFSCA and obtaining results of examples presented in the paper are public available for download in^24^.

### Centre-of-pressure fluctuations of postural sway

We apply the proposed method to the human centre-of-pressure (CoP) trajectories in a standing posture^25^. The CoP is the projection of the centroid of the vertical force distribution on the ground plane. The CoP characteristics have been studied extensively to evaluate balance dysfunction for patients with neurological and motor disorders and to understand the postural control mechanism^26,27^.

To extract the oriented scaling components from the CoP characteristics, we analysed CoP trajectories measured in two standing posture conditions: quiet standing with eyes open (Fig. 4a) and a voluntary sway movement from the steady position to right-backward position with eyes open (Fig. 5a); the recording duration was 30 s with a sampling frequency of 100 Hz (for more details of the measurement, see^28,29^). Recordings of CoP trajectories were obtained from a project approved by the Institutional Review Board of the National University of Physical Education and Sport of Ukraine. The procedures complied with the Declaration of Helsinki regarding human experimentation. Written informed consent was obtained from all the participants prior to the test.

The angle dependence of $F^{(\theta )} (\tilde{s})$ was evaluated over the range of $0 \le \theta < \pi$ in increments of $\pi /64$ rad (Figs. 4b,c, 5b,c). We set the scaling range $1.2< \log _{10} \tilde{s} < 2.5$ (from 0.16 to 3.2 s) and estimated the slopes of linear regressions (Fig. 4d) to find two representative orientations.

In the quiet standing condition, the minimum and maximum slopes were observed at $\hat{\theta }_{\mathrm{min}} = 6^{\circ }$ and $\hat{\theta }_{\mathrm{max}} = 124^{\circ }$, respectively (Fig. 4d). Thus, the estimated orientations were $\hat{\theta }_{1} = 96^{\circ }$ and $\theta _{\mathrm{max}} = 34^{\circ }$. As shown in Fig. 4d, the estimated minimum and maximum slopes (1.92 and 2.07, respectively) were almost equal. Nevertheless, the scaling behaviours of the decomposed components $\left\{ \hat{\epsilon }_1 [i] \right\}$ and $\left\{ \hat{\epsilon }_2 [i] \right\}$ (Fig. 4e) showed a nontrivial difference. As shown in Fig. 4f, a crossover point around $\log _{10} \tilde{s} = 2.2$ was observed only in the component $\hat{\epsilon }_2$ with an orientation of $34^{\circ }$. The observed scaling exponent $\alpha \approx 2.0$ indicates an fBm-like behaviour with long-range-correlated increments, which suggests a rather anomalously expanding and unstable CoP trajectory. In contrast, the smaller scaling exponent ($< 1.0$) observed at the larger scales in the $\hat{\epsilon }_2$ orientation suggests a bounded and stable CoP trajectory. Therefore, the $\hat{\epsilon }_2$ orientation would correspond to the most stable direction of the posture control. This result is reasonable from an anatomical point of view, as a standing human body has more stability against left-right oscillations than against forward-backward oscillations while attempting to stand still. The $\hat{\epsilon }_1$ orientation almost coincided with the face-front (anteroposterior) direction, whereas the $\hat{\epsilon }_2$ orientation was considerably deviated from the shoulder-width (mediolateral) direction. This deviation originates from the motor asymmetry of the lower limbs. The mean CoP position of the subject was shifted slightly to the right from the coordinate centre of the platform, i.e. the subject body weight was distributed more on his dominant right leg than on his left one. Hence, we conclude that the subject controlled the postural balance mainly with his right foot^30^.

In the condition of voluntary sway movement, the subject was asked to perform swaying movements in the backward-right direction (Fig. 5a). In this condition, the minimum and maximum slopes were observed at $\hat{\theta }_{\mathrm{min}} = 37^{\circ }$ and $\hat{\theta }_{\mathrm{max}} = 132^{\circ }$, respectively (Fig. 5d). Thus, the estimated orientations were $\hat{\theta }_{1} = 127^{\circ }$ and $\theta _{\mathrm{max}} = 42^{\circ }$. The orientation with $\hat{\theta }_{1}$ almost coincided with the body sway direction, and the orientation with $\hat{\theta }_{2}$ was almost perpendicular to it. The scaling exponent of $\hat{\epsilon }_1$ along the body sway direction was 2.5 (Fig. 5f), indicating a smoothly spreading fluctuation. In contrast, the scaling exponent of $\hat{\epsilon }_2$ at larger scales ($> 1$ s) was 1.5 (Fig. 5f), indicating a Brownian-motion-like fluctuation.

The decomposed components $\hat{\epsilon }_1$ and $\hat{\epsilon }_2$ (Figs. 4e and 5e) appeared to be moving along each axis in an independent manner. Moreover, the cross-correlations between $\hat{\epsilon }_1$ and $\hat{\epsilon }_2$ were almost zero in both conditions. The Pearson cross-correlation coefficients were $-0.07$ and $-0.12$, respectively, in the quiet standing and leaning motion conditions. Hence, our approach could detect and reconstruct the reasonable main orientations in the posture control dynamics. However, the kinetic and mathematical mechanism generating the observed fluctuations is still unclear.

**Figure 7:**
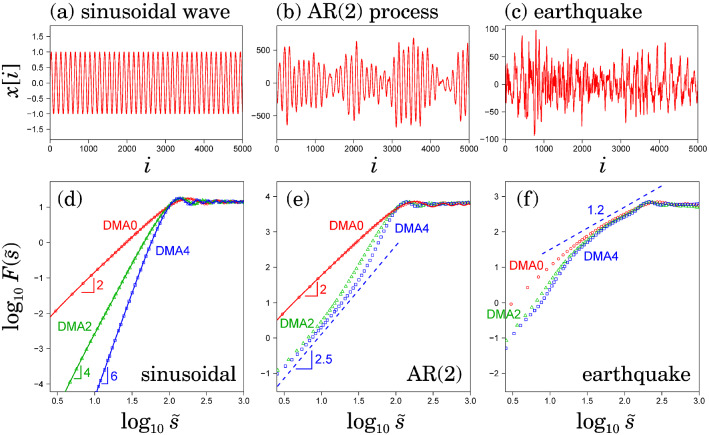
DMA results for oscillating time-series. (**a**) Sinusoidal wave. (**b**) Second-order auto-regressive (AR(2)) model. (**c**) Earthquake ground acceleration of the aftershock (Fig. 6). The slopes ($\approx 1.2$) in the range smaller than the plateau regime characterise the correlation properties of the noisy fluctuations.

### Great 2011 Tohoku-oki earthquake

The second application is the analysis of the largest earthquake on record in Japan and the fourth largest earthquake in the world from 1900 to 2020. The Tohoku-oki earthquake occurred at 14:46 JST (05:46 UTC) on 11 March 2011 in Japan. It was a magnitude 9.0 undersea mega-thrust earthquake off the coast of Japan^31^. It induced a giant tsunami with waves over tens of meters high, which caused serious nuclear power plant accidents. The time evolution of the main earthquake lasted for approximately 150 s and was associated with a fault rupture 440 km long in a north–south direction and 180 km wide along the plate interface. The source of the earthquake was not a single large earthquake but several interdependent earthquakes linked to each other.

We analyse the recordings^32^ of a seismic sensor located at Shin-Machi, Wakuya-cho, Toda-gun Miyagi Prefecture (Latitude: 38.5401$^{\circ }$N, Longitude: 141.1272$^{\circ }$E) to characterise the complicated earthquake process. From this location, the epicentre was located approximately 111 km east and the earthquake sources were distributed over the azimuth angle range of approximately $102^{\circ }$ to $140^{\circ }$, where the azimuth angle $\phi$ is defined as the angle from the north direction counted clockwise, (i.e. $\phi \approx \pi /2 - \theta$). The analysed 2D time series is the acceleration on the ground surface with the north–south (NS) and east–west (EW) axes at the sampling frequency of 100 Hz. The ground acceleration has been considered the most important factor in determining the stress induced to structures during earthquakes. The top two panels in Fig. 6 show 3-min time series. This time series could be separated into four representative phases: P-, first S-, second S-, and aftershock waves (Fig. 6a,b). The previous studies^31^ using inversion analysis have identified three main sources generating the representative waveforms. The first and second S-waves were generated, respectively, by the first and second main sources in the east–southeast direction from the measurement point. In addition, even in the aftershock phase, the earthquake wave induced by the third main source in the south–southeast direction was overlapped. However, the corresponding S-wave was not dominant because of the distance decay effect.

In each phase, the angle dependence of $F^{(\theta )} (\tilde{s})$ was estimated over the range of $0 \le \theta < \pi$ in increments of $\pi /64$ rad using fourth-order DDMA. $F^{(\theta )} (\tilde{s})$ and the local slopes are shown in Fig. 6c,d, together with the estimated representative orientations (red and blue dashed lines). We set the scaling range $1.3< \log _{10} \tilde{s} < 2.2$ (from 0.20 to 1.6 s) and estimate the slopes in this range using linear regressions (Fig. 6e) to find two representative orientations.

A remarkable transition was observed in the orientations and the correlation properties before and after the arrival of the first S-wave. In the P-wave phase, the estimated orientations of $\hat{\epsilon }_1$ and $\hat{\epsilon }_2$ were $\hat{\theta }_1 \approx 85^{\circ }$ and $\hat{\theta }_2 \approx 170^{\circ }$, respectively (the first column in Fig. 6 c,d). The corresponding slopes in the range of $1.3< \log _{10} s < 2.2$ were close to 0.5 and less than 1.0 (Fig. 6e). The fluctuation functions showed an evident difference between $\hat{\epsilon }_1$ and $\hat{\epsilon }_2$ (Fig. 6f). A change in the orientations was observed immediately after the arrival of the first S-wave (the second column in Fig. 6c,d). In the first and second S-waves, the orientations of $\hat{\epsilon }_1$ and $\hat{\epsilon }_2$ were $\hat{\theta }_1 \approx 135^{\circ }$ and $\hat{\theta }_2 \approx 45^{\circ }$, respectively. In the aftershock wave, the orientations were rotated approximately $20^{\circ }$ in the counter clockwise (the fourth column in Fig. 6c,d). In addition, gradual increases of the slopes in the range of $1.3< \log _{10} \tilde{s} < 2.2$ were observed through the earthquake process (Fig. 6f). The slopes approached 1.5 through and after the second S-wave phase.

All the plots of the fluctuation functions (Fig. 6f) showed a (non-increasing) plateau in the range of $\log _{10} \tilde{s} > 2.1$, indicating oscillating behaviour. To illustrate such behaviour, we also analysed the numerical time-series of a purely sinusoidal wave (Fig. 7a) and a second-order auto-regressive (AR(2)) model (Fig. 7b) using DMA. Here, the periods in the sinusoidal wave and the AR(2) model are set to $10^{2.1}$ points. In Fig. 7d, the plot of $\log _{10} F^{(\theta )}(\tilde{s})$ vs. $\log _{10} \tilde{s}$ shows a power-law increase in scales shorter than the oscillating period ($\log _{10} \tilde{s} < 2.1$) and a plateau in scales longer than the oscillating period. Notably, the power-law (scaling) exponents of the purely sinusoidal wave (Fig. 7d) cannot be linked with the power-law correlation properties and the exponent depends on the order of the DMA. In contrast, the AR(2) model (Fig. 7b) shows a short-term power-law correlation characterised by $\alpha =2.5$ (dashed lines in Fig. 7e) because of the power-law of the power spectrum as $S(f) \sim f^{-4}$ for the high-frequency range. However, as shown in Fig. 7d, zeroth-order DMA cannot detect a scaling exponent larger than 2^6^. That is, zeroth-order DMA (commonly called DMA so far) cannot distinguish between a purely sinusoidal wave (Fig. 7a) and an AR(2) model. Therefore, the consistency of the scaling behaviour needs to be tested using higher-order DMAs to detect meaningful scaling behaviour. In our earthquake analysis (Fig. 7c,f), the scaling behaviour in the range of $\log _{10} \tilde{s} > 1.3$ in higher-order DMAs showed consistent results.

The observed transition before and after the arrival of the first S-wave would reflect the mechanism difference. The P-wave is a compressional wave with no shear components and it travels faster than other seismic waves. In contrast, the S-wave is a shear wave and propagates faster than the P-wave. Although a detailed interpretation of our observation is not possible at present, our approach would provide a new method for earthquake analysis and advance the existing earthquake research activities, such as Refs.^33–35^.

## Discussion

We have proposed a sum of mixed fluctuations with different orientations and fractal scaling features as a model for anisotropic 2D trajectories. A decomposition method called OFSCA was developed to decompose a 2D trajectory into the original oriented components. Through the analysis of a sample anisotropic 2D fGn model, we have demonstrated that the OFSCA estimates the model parameters (the orientation angle of each component and its scaling parameter) successfully. Moreover, the analysis of real-world anisotropic 2D trajectories, such as human CoP and the great 2011 Tohoku-oki earthquake, has demonstrated the existence of their oriented fractal scaling components and provided detailed characterisation of their properties. These findings are merely exploratory, and further studies of the oriented scaling components will provide new insights on the complex fluctuations.

Our observations on earthquake data analysis based on OFSCA are consistent with the findings of Ref.^31^, where the motion characteristics of the tectonic movement during the Tohoku earthquake were studied using inverted teleseismic P- wave data. The authors showed the relationship between slip distribution (tectonic movements) and strong ground motion while considering a long slip duration, large stress drop, and extensional (normal faulting) aftershocks. We consider that there could be a relationship between the Hurst exponent and strong ground motion, since as discussed in the Ref.^36^ the power law in the earthquake statistics and non-linear nature of the earthquake propagation comes from the fractal nature of rough crack surfaces of crust and tectonic plate dynamics. We assume that OFSCA approach might provide basic information about the rupture process for real-time monitoring (real-time seismology). Further application of OFSCA to seismic data and the interpretation of the fluctuation characteristics of tectonic movements are required to understand the earthquakes more deeply and to predict their occurrence and propagation in the future.

Further extension of OFSCA is feasible for postural sway analysis, which has been used for the medical diagnosis and prognosis of patients with neurological and motor disorders. When a subject has a motor/sensory disorder (such as hemiplegia or incomplete spinal cord injury), which has an asymmetrical effect on the standing stability^37^, the evaluation of the directional dependency of the CoP trajectory could provide useful information on the stabilising ability and dysfunction.

The proposed OFSCA can be used to characterise other real-world 2D trajectories. For instance, in Ref.^38^, an automated continuous monitoring system for tracking the behaviour of pigs was used to monitor their movements with a depth camera. Then, using 2D trajectories of pig movement, the directional behaviour and characteristics of the locomotion dynamics could be quantified with OFSCA and mapped onto the animal health issues to detect warning signs. This approach can be used to characterise the trajectories of other animals, such as fish, cows, and unicellular organisms. It can also be applied in several other behavioural, ecological, and veterinarian studies, and to amend the existing techniques of 2D and 3D trajectory analyses^39,40^.

Another promising application is the analysis of the 2D trajectories of individuals encountered in group games, such as soccer, in both humans^41^ and robots^42^. The group dynamics and interconnections of the player’s movement dynamics may provide interesting insights for sports studies, and the orientation of the fluctuation characteristics can be a valuable feature of single, group, and event-specific trajectories^43^. Furthermore, the OFSCA approach can be extended to higher-dimensional data analysis, e.g. studying complex evolutionary dynamics^44^, or understanding the structure and patterns in high-dimensional RNA sequencing datasets^45^.
